# How to find RNA thermometers

**DOI:** 10.3389/fcimb.2014.00132

**Published:** 2014-09-18

**Authors:** Francesco Righetti, Franz Narberhaus

**Affiliations:** Microbial Biology, Ruhr University BochumBochum, Germany

**Keywords:** regulatory RNA, RNA structure, heat shock, virulence, next-generation sequencing

## Abstract

Temperature is one of the decisive signals that a mammalian pathogen has entered its warm-blooded host. Among the many ways to register temperature changes, bacteria often use temperature-modulated structures in the untranslated region of mRNAs. In this article, we describe how such RNA thermometers (RNATs) have been discovered one by one upstream of heat shock and virulence genes in the past, and how next-generation sequencing approaches are able to reveal novel temperature-responsive RNA structures on a global scale.

## How RNA thermometers work

**Figure 1:**
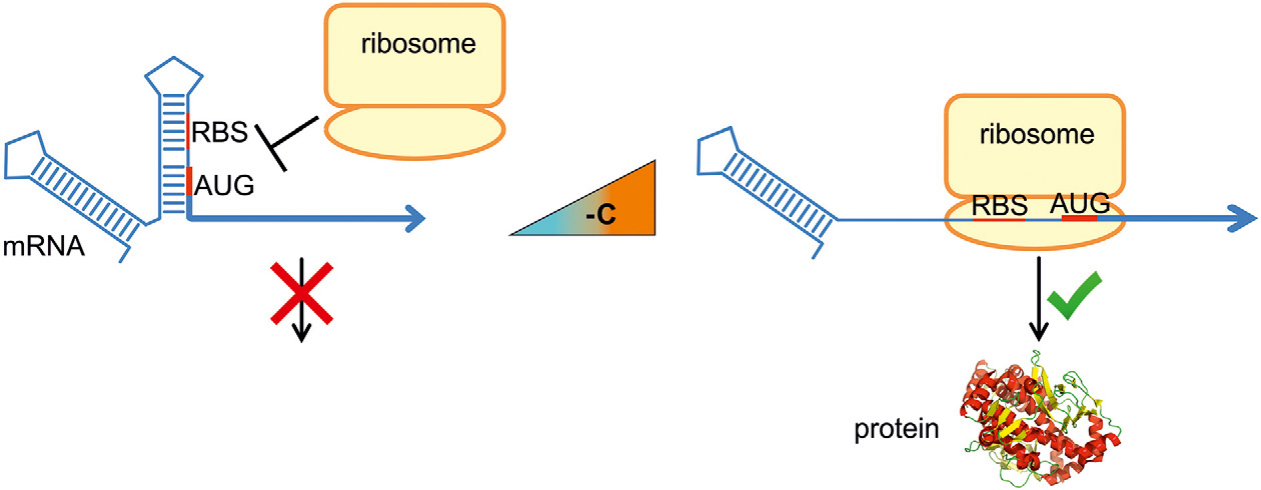
**RNA thermometer-mediated translational regulation**. RNA thermometers trap the ribosome binding site (RBS) and/or the translation initiation codon (AUG) of an mRNA by base pairing within a secondary structure. An increase in temperature to 37°C (virulence genes) or 40–42°C (heat shock genes) destabilizes the structure in a reversible, zipper-like manner. Liberation of the RBS permits formation of the translation initiation complex and translation occurs.

RNA molecules are not linear, but fold into complex three-dimensional structures. Base pairing of proximal nucleotides generates secondary structures, like stem-loops. Long distance interactions allow formation of tertiary structures, like pseudoknots or kissing loops. It is well established that the structure is fundamental for the biological function of non-coding RNA such as tRNA and rRNA. What is now emerging is that the three-dimensional architecture of mRNA influences its entire life cycle: transcription, maturation, translation and degradation. RNA-mediated gene regulation is relevant as it is fast and energy-saving because it bypasses the expression of transcription factors. Structured RNA elements are known to respond to different stimuli, for example metabolite-sensing riboswitches (Serganov and Nudler, 2013). RNA thermometers (RNATs) modulate translation efficiency of an mRNA according to the ambient temperature (Kortmann and Narberhaus, 2012). They are usually located in the 5′-untranslated region (5′-UTR) of an mRNA and form a base-paired structure that involves the ribosome binding site (RBS) and/or the translation initiation codon (Figure 1). Increasing or decreasing temperatures alter the conformation of that structure, allowing or preventing ribosome access and thus translation. RNAT have been localized not only at the 5′-end of a transcript but also in intercistronic regions, where they differentially control gene expression (Krajewski and Narberhaus, 2014). The instantaneous response makes RNAT suitable for regulation of heat shock and virulence genes. The reversibility of the melting process permits simple bidirectional control of translation because the structure melts open and allows translation while the temperature increases, but refolds and blocks translation when the temperature drops again (Chowdhury et al., 2003; Kortmann et al., 2011).

In contrast to fairly conserved ligand-binding riboswitches, the structural elements found in RNATs show little sequence conservation, if any (Kortmann and Narberhaus, 2012). Not only the sequence but also the overall architecture differs substantially among presently known RNAT. A single short stem-loop structure can be sufficient to confer thermoregulation, as shown for the 46-nt long *hsp17* RNAT from *Synechocystis* sp. PCC 6803 (Kortmann et al., 2011). Other RNAT, like the ROSE (Repression Of heat-Shock gene Expression) element, have a very complex structure ranging from two to four stem-loops (Nocker et al., 2001a,b; Chowdhury et al., 2003; Waldminghaus et al., 2005; Krajewski et al., 2013). Temperature sensing is based on several non-canonical, heat-labile base pairs (Chowdhury et al., 2006). Another poorly conserved RNAT class is characterized by four consecutive uridines that base-pair with the RBS. In the best characterized representative, the *Salmonella enterica* FourU thermometer of the small heat shock gene *agsA*, Mg^2+^ ions play a crucial role in stabilization of the closed conformation at low temperature (Waldminghaus et al., 2007b; Rinnenthal et al., 2010, 2011).

Long and complex structures involving regions in both the untranslated and coding regions are used to permit translation at low temperature. The *E. coli cspA* transcript adopts two mutually exclusive conformations at 37 and 10°C. The conformational switch affects the translatability and stability of the mRNA resulting in massive induction of the cold shock protein CspA at low temperatures (Yamanaka et al., 1999; Giuliodori et al., 2010).

In this article, we briefly recapitulate how RNATs have been discovered in the past, before we go on to discuss the potential of recently established next-generation sequencing techniques for genome-wide identification of new regulatory RNA elements.

## Serendipitous discovery of RNA thermometers

The first reported RNAT are unique and rather complex. Their discovery has been preceded by decade-long research on phage λ and the *Escherichia coli* heat shock response. The 5′-UTR of the λ cIII transcript forms two mutually exclusive conformations whose equilibrium strictly depends on the temperature (Altuvia et al., 1989). At temperatures below 37°C, the RNA adopts a conformation, in which the RBS is accessible and translation occurs. At a higher temperature (45°C), the equilibrium shifts toward the conformation that partially occludes the RBS. Low levels of cIII in turn initiate the lytic cycle of phage λ. An RNAT that liberates the SD sequence with increasing temperature was first found in the *E. coli rpoH* gene encoding the alternative sigma factor σ^32^, the master regulator of the heat shock response. The temperature-sensing structure is very complex and involves the 5′-UTR and up to 229 nucleotides of the coding region (Morita et al., 1999a,b).

These seminal findings along with quantitative studies on the role of secondary structures in translation initiation (De Smit and Van Duin, 1990) established the concept that temperature-dependent modulation of RNA structures can regulate translation efficiency. Since then numerous RNAT-controlled heat shock and virulence genes have been discovered. Probably, the most abundant class of RNAT is the ROSE family, always associated with bacterial small heat shock genes. The first ROSE element was found upstream of the *Bradyrhizobium japonicum* heat shock protein A gene (*hspA*) (Nocker et al., 2001a,b). Conserved nucleotides upstream of the open reading frame were first believed to serve as binding site for a temperature-responsive transcription factor (Narberhaus et al., 1998) but later shown to be involved in RNA structure formation (Nocker et al., 2001a). *In silico* prediction of the RNA structure and reporter gene fusions (Nocker et al., 2001a; Chowdhury et al., 2003), CD spectroscopy (Chowdhury et al., 2003), and NMR (Chowdhury et al., 2006) helped to understand the underlying RNA-based control mechanism.

Almost simultaneously, the first RNAT-regulated virulence gene was described in *Listeria monocytogenes* (Johansson et al., 2002). The *prfA* gene encodes a transcription factor that controls the synthesis of a number of important virulence factors. A hairpin structure in its 5′-UTR partially masks the ribosome binding region and permits efficient translation only at host body temperature. The *prfA* thermosensor is peculiar as it integrates not only the temperature signal by structural changes but also metabolic information via a riboswitch-derived small regulatory RNA (Loh et al., 2009).

Only recently, three new RNATs helping to escape the human immune system at increasing temperature were identified in *Neisseria meningitidis* (Loh et al., 2013). Several *N. meningitidis* strains resistant to complement-mediated killing were found to contain an 8-nucleotide deletion upstream of the *css* operon causing increased production of CssA involved in capsule production. After establishing that 38 transcription factors did not control *cssA* expression, it emerged that an RNA structure is responsible for this effect. At low temperature, the 5′-UTR assumes a hairpin structure that prevents ribosome binding. This structure is much weaker in the mutated RNA. Two other *Neisseria* genes involved in immune escape are regulated by RNATs. They encode the factor H binding protein and Lst involved in lipopolysaccharide modification.

## Guilty by association: heat shock and virulence genes

The growing awareness of temperature-responsive RNA structures triggered systematic searches for RNATs upstream of heat shock and virulence genes. Computational predictions by mfold (Zuker, 2003) or other programs (Seetin and Mathews, 2012) can be used to locate RNA structures in the 5′-UTR of selected genes or in entire genomes (Waldminghaus et al., 2007a). Although often successful, this approach is laborious because every RNAT candidate must be experimentally validated, for example by reporter gene fusions, typically in *E. coli* (Klinkert et al., 2012), and by site-directed mutagenesis, structure probing and toeprinting analysis.

The most common genes subjected to RNAT-mediated regulation code for small heat shock proteins (sHSPs), ATP-independent chaperones that maintain client proteins in a folding-competent state. The systematic search for potential RNATs upstream of sHSP genes has led to the identification of more than 40 ROSE-type RNATs in diverse alpha- and gamma-proteobacteria (Waldminghaus et al., 2005). Structurally very diverse RNATs control sHSP genes in cyanobacteria. A short single-hairpin structure is sufficient to confer thermoregulation to *hsp17* of *Synechocystis sp*. PCC 6803 (Kortmann et al., 2011). *Anabaena variabilis* encodes two sHSPs. One is controlled by a short RNAT that primarily blocks the start codon, the other controls access to the SD sequence by a ROSE-like sequence and contains an additional extended hairpin structure that might be involved in tertiary RNA-RNA interactions (Cimdins et al., 2014). This study also provided evidence for a complex RNA structure controlling translation of the *hspA* gene of *Thermosynechococcus elongatus*, a thermophilic cyanobacterium with an optimal growth temperature at 57°C.

The founding member of the fourU class of RNATs was found to control the *Salmonella* sHSP gene *agsA* (Waldminghaus et al., 2007b). These RNATs are characterized by a stretch of four uridines that pair with the AGGA sequence of the SD region. Other members of this class control synthesis of the periplasmic protease HtrA in *Salmonella* (Klinkert et al., 2012), of the virulence regulator LcrF (VirF) in *Yersinia pestis* (Hoe and Goguen, 1993) and *Yersinia pseudotuberculosis* (Böhme et al., 2012), and of the heme receptor proteins ChuA or ShuA in pathogenic *E. coli* strains and in *Shigella dysenteriae*, respectively (Kouse et al., 2013).

There is accumulating evidence that various other heat shock and virulence genes are under control of gene-specific RNATs. Differential regulation of individual genes in poly-cistronic heat shock operons is achieved by an RNAT upstream of *groES* in the *Salmonella groESL* operon (Cimdins et al., 2013) and two unrelated RNATs upstream of *hspX* and *hspY* in the *Pseudomonas putida hspXYZ* operon (Krajewski et al., 2014). Two identical RNATs were found upstream of the *Leptospira interrogans* virulence genes *ligA* and *ligB* coding for putative lipoproteins important for adhesion and complement resistance (Matsunaga et al., 2013).

Restricting searches for novel RNATs to regions upstream of annotated heat shock and virulence genes is unlikely to reveal RNATs in unexpected places. New bioinformatic tools, such as RNAtips (temperature-induced perturbation of structure) (Chursov et al., 2013), RNAthermsw (Churkin et al., 2014) or other programs able to predict transient RNA structures (Zhu et al., 2013) may help reveal temperature-responsive RNA structures on a genome-wide scale. However, while computational methods are advanced enough to accurately predict short and stable secondary structures, their reliability decreases substantially with increasing length of the RNA molecule or when complex structures, such as pseudoknots and other tertiary interactions, come into play. Therefore, unbiased experimental high-throughput approaches are desirable for the identification of regulatory RNA structures on a global scale.

## The RNA structurome revealed by next-generation sequencing

The genome-wide assessment of RNA structures relies on structure-probing techniques able to distinguish single- and double-stranded regions. Traditional structure-probing experiments are based on the *in vitro* treatment of a single RNA species with a variety of chemical or enzymatic probes, capable to modify or cut selectively paired or unpaired nucleotides (Ehresmann et al., 1987; Weeks, 2010). Several chemical reagents modify or cleave unpaired or flexible bases, such as Pb^2+^ (Gornicki et al., 1989), dimethyl sulfide (DMS), *N*-methylisatoic anhydride (NMIA), kethoxal, and 1-cyclohexyl-(2-morpholinoethyl)carbodiimide metho-p-toluene sulphonate (CMCT). Enzymatic single-strand specific probes include nucleases S1, A, T1, I. The RNase V1 enzyme cleaves at double-stranded nucleotides. Hydroxyl radicals cleave at RNA bases that are solvent-exposed. Upon treatment, modified or cut positions are mapped by polyacrylamide gel electrophoresis, if necessary after reverse transcription. These approaches are well established and were successfully applied in the structural analysis of a wide range of RNA molecules.

RNA secondary structure can also be sampled *in vivo*. Chemicals that quickly penetrate the cell membrane, such as Pb^2+^ (Lindell et al., 2002, 2005) and DMS (Wells et al., 2000; Liebeg and Waldsich, 2009), can be employed to probe the intracellular RNA structure. The *in vitro* conformation of some RNAs can differ from its functional conformation *in vivo*, due to the different solvent conditions and the presence of ligands, proteins or other RNAs that can bind to the RNA and alter its architecture (Zemora and Waldsich, 2010). Another important aspect is that the folding process of a nascent bacterial RNA is coupled to its transcription and translation. Cotranscriptional folding and the progressive binding of proteins and ribosomes guide the folding into secondary structures that can differ from the structure of an RNA molecule folded and probed *in vitro*. *In vivo* structure probing averages the structural state of each nucleotide from all conformations the RNA molecule adopts during its life cycle. Together, *in vivo* and *in vitro* data provide valuable complementary information to unveil biologically relevant structures and their dynamics.

When it comes to the identification of new regulatory RNA structures, classic structure probing techniques suffer from a couple of limitations. First, only a single species of RNA can be tested per experiment, making this technique suitable for the validation of individual structures but not for global screening purposes. Second, only a relatively short region of several 100 nucleotides of an *in vitro* synthesized and labeled RNA can be investigated. The selective 2′-hydroxyl acylation analyzed by primer extension (SHAPE) approach takes advantage of capillary electrophoresis of cDNA obtained from RNA treated with NMIA, or its derivatives (Merino et al., 2005). NMIA attacks flexible (unpaired) bases and forms 2-O adducts that terminate reverse transcription. SHAPE could successfully probe the structure of the 9-kb HIV RNA genome leading to the identification of structural regions that interact with nucleocapsid proteins and elements important for the regulation of viral gene expression (Watts et al., 2009). The development of new chemical probes allowed the application of the SHAPE protocol to the analysis of RNA structure within living cells (Spitale et al., 2013).

Only recently high-throughput sequencing technologies have been successfully applied to RNA structure probing in order to obtain experimentally-derived genome-wide insights into RNA folding. The global landscape of the structural organization of a whole transcriptome of an organism has been referred as the “RNA structurome” (Wan et al., 2011). In this case, a complex RNA population is cleaved or modified with structure-specific probes prior to cDNA synthesis and sequencing. The sequencing reads are mapped to the reference genome or transcriptome and the position of each read along the transcript provides information on single- and double-stranded nucleotides. Structural data can be used to constrain RNA structure prediction algorithms in order to obtain more accurate experimentally-derived secondary structure models of all the sequenced transcripts. The bioinformatic analysis of the raw data is a challenge and considerable effort has been put on software development in order to facilitate data interpretation (Aviran et al., 2011; Ouyang et al., 2013; Zhong and Zhang, 2014).

**Figure 2:**
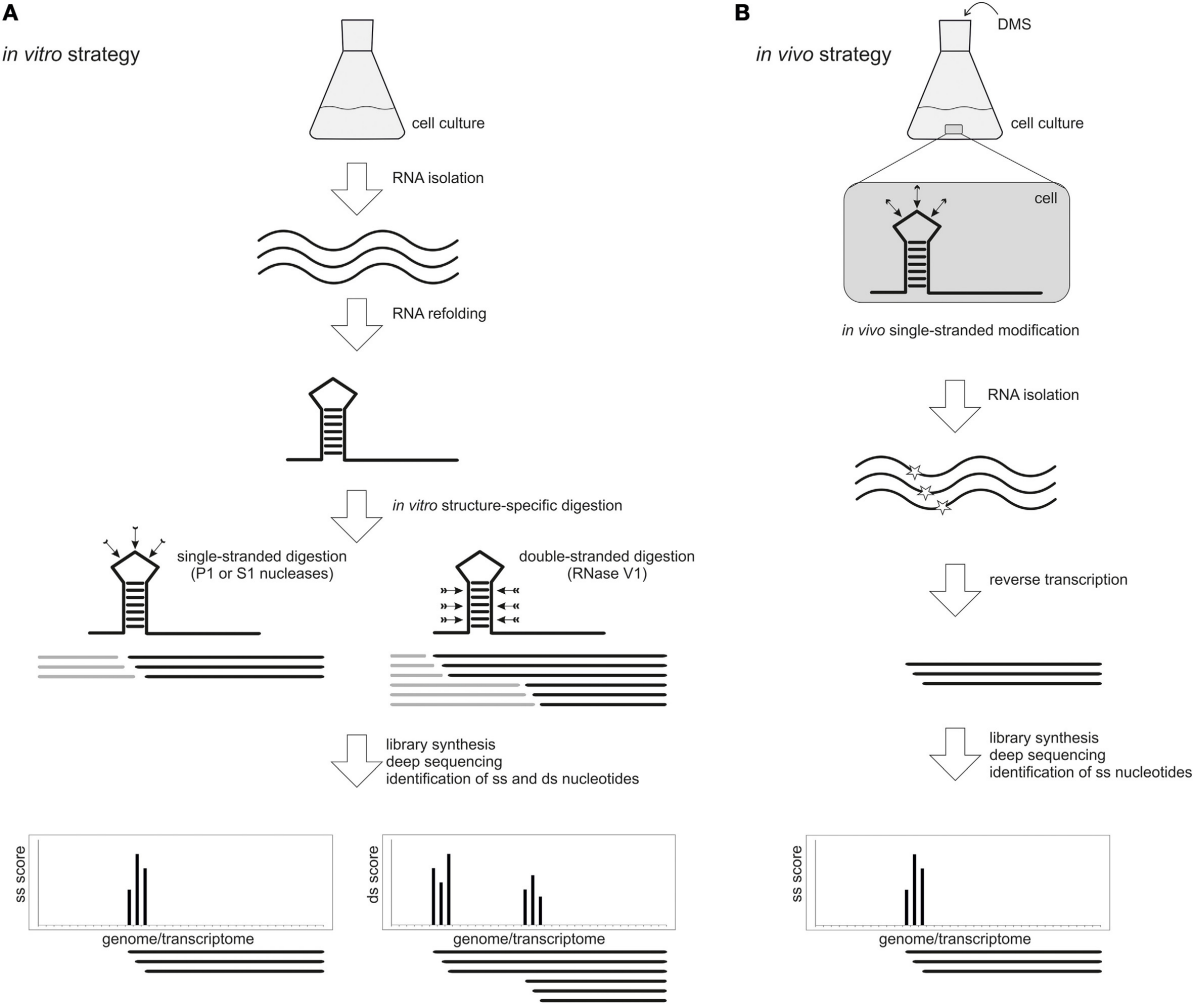
**High-throughput genome-wide RNA structure probing. (A)** In an *in vitro* approach, RNA is isolated from a cell culture and re-folded prior to treatment with single-stranded (ss) or double-stranded (ds) specific probes like nucleases P1 and S1 or RNase V1, respectively. After library preparation and deep sequencing, the resulting reads are mapped to the reference genome or transcriptome. The score at each nucleotide indicates whether it is in a single-stranded (ss) or double-stranded (ds) conformation. **(B)** The *in vivo* approach allows to probe native RNA structures directly inside the cell using chemical probes that penetrate the membranes and modify nucleotides in a ss conformation. A widely used probe is DMS, which methylates unpaired adenine and cytosine bases. After DMS treatment, the RNA is isolated and the modification position is detected by reverse transcription and deep sequencing. This approach permits the identification of ss regions only.

The first pioneering studies took advantage of structure-specific enzymatic probes. Two examples are the PARS (Parallel Analysis of RNA Structure) (Kertesz et al., 2010) and the Frag-seq (Fragmentation Sequencing) (Underwood et al., 2010) approaches that were first applied to the analysis of the yeast *Saccharomyces cerevisiae* poly(A) transcriptome and mice nuclear transcriptome, respectively (Figure 2A). In a typical PARS experiment, poly(A) enriched total RNA is isolated, refolded *in vitro* and partially digested either with the single-strand specific nuclease S1 or the double-strand specific RNaseV1. Paired and unpaired regions of the whole transcriptome are deduced from a comparison of the two sequenced libraries. This approach unveiled the secondary structure profile of more than 3000 yeast transcripts and revealed interesting structural features, such as a higher average secondary structure occurrence in coding regions compared to untranslated regions, a three-nucleotide periodicity of secondary structure across coding regions and correlation between translation efficiency and the structure around the translation start site (Kertesz et al., 2010). Most recently, the PARS approach was applied to the human transcriptome (Wan et al., 2014). Beside the structural features that demarcate coding regions, splicing junctions and microRNA binding site, the authors could identify over 1900 single nucleotide variants that alter the local RNA structure. These variations can affect gene expression and microRNA and protein binding to the RNA molecule.

Purified nuclear RNA from mice, which was refolded *in vitro* and partially digested with the single-strand specific nuclease P1, has been analyzed by Frag-seq. This approach could successfully confirm single-strand regions of non-coding RNA with known structure and unveiled the secondary structure of non-coding RNA with previously unknown structure (Underwood et al., 2010). In a SHAPE-seq pipeline, the SHAPE method was coupled to high-throughput sequencing to simultaneously read out the structures of a mixture of 7 different RNA molecules (Lucks et al., 2011). Recently, the whole RNA structuromes of *Arabidopsis thaliana* (Zheng et al., 2010; Li et al., 2012b), *Drosophila melanogaster* and *Caenorhabditis elegans* (Li et al., 2012a) have been analyzed by coupling nuclease digestion and high-throughput sequencing. Such global RNA folding profiles allow the identification of structural features involved in RNA-related processes, such as translation regulation, splicing and microRNA-mediated regulation.

It is easily conceivable that temperature-responsive structures such as RNATs can be discovered by this technology and a first step in this direction has been undertaken by probing the whole yeast transcriptome at different temperatures (Wan et al., 2012). The melting temperature of each sequenced transcript was measured at a single nucleotide resolution, which led to the identification of RNA regions that undergo conformational changes in a physiological range of temperature. These regions might have a role in temperature-mediated post-transcriptional gene regulation.

*In vivo* global structure probing strategies have been attempted only very recently and applied to *A. thaliana* seedlings (Ding et al., 2014), *S. cerevisiae* and mammalian cells (Rouskin et al., 2014) and *S. cerevisiae* (Talkish et al., 2014) (Figure 2B). All these approaches were based on the treatment of living cells with DMS, a chemical probe that can quickly cross the membranes and modify preferentially unpaired adenine and cytosine residues. Modified bases block reverse transcription and sequencing of the resulting fragments permits the identification of the reactive sites and the mapping of single-stranded nucleotides. Comparison between *in vitro* and *in vivo* data will ultimately provide a detailed picture of the RNA structurome in its physiological context.

## Conclusion

RNA structure discovery has entered a new era by coupling structure probing to next-generation sequencing. Quite surprisingly, no prokaryotic RNA structurome has been reported so far. Given that the principle has been established in yeast (Wan et al., 2012), it is likely that it will be used to identify temperature-sensitive RNA elements in bacteria in the near future. It will be particularly interesting to learn whether RNATs exist in other places than heat shock and virulence gene transcripts.

### Conflict of interest statement

The authors declare that the research was conducted in the absence of any commercial or financial relationships that could be construed as a potential conflict of interest.
